# Frequency of periodontal disease in head and neck cancer patients after radiation therapy: a cross-sectional study

**DOI:** 10.1186/s12903-025-06391-7

**Published:** 2025-07-02

**Authors:** Asmaa Abou-Bakr, Enji Ahmed, Hany William, Fatma E. A. Hassanein

**Affiliations:** 1 Oral Medicine and Periodontology, Faculty of Dentistry, Galala University, Suez, Egypt; 2 Oral Medicine and Periodontology, Faculty of Dentistry, Cairo University, Giza, Egypt; 3 Radiation Oncology, Ahmed Maher Teaching Hospital, Cairo, Egypt; 4 Oral Medicine, Periodontology, and Oral Diagnosis, Faculty of Dentistry, King Salman International University, El-tor, Egypt

**Keywords:** Egypt, Cancer, Saliva, Periodontitis, Head and neck cancer, Radiotherapy

## Abstract

**Background:**

Patients with head and neck cancer (HNC) receiving radiation therapy (RT) have complications affecting both general and dental health. While RT is effective against HNC, it negatively impacts oral tissues, including changes in periodontal attachment. This study aimed to evaluate the frequency of periodontal disease among HNC patients after RT in a sample of the Egyptian population, as well as to assess the associated risk factors.

**Materials and methods:**

A prospective cross-sectional study was conducted on 189 HNC patients attending a radiation center in Cairo, Egypt. Eligible patients underwent a full periodontal examination including plaque index (PI), bleeding on probing (BOP), clinical attachment level (CAL), and probing pocket depth (PPD). Subsequently, Clinical Oral Dryness Score (CODS), salivary flow rate and body mass index (BMI) were assessed to examine associations with periodontal health.

**Results:**

Periodontal disease was diagnosed in 183 (96.8%) HNC patients. Among them, 174 (95.08%) cases were diagnosed as periodontitis and 9 (4.9%) as gingivitis. The distribution of periodontitis different stages was as follows: Stage I was found in 21 (12.07%) cases, Stage II in 30 (17.24%), Stage III in 55 (31.61%), and Stage IV in 68 (39.08%). The correlation between the number of RT fractions and periodontal disease severity was not statistically significant (*p* = 0.837). However, there was a strong negative correlation between periodontitis stage and both body mass index (BMI) and salivary flow rate. In contrast, strong positive correlations were observed between periodontitis severity and RT dose, plaque percentage, and Clinical Oral Dryness Score (CODS).

**Conclusions:**

The frequency of periodontitis in the studied sample of HNC patients post RT was 95.08%, reflecting a notably high burden. The most prevalent periodontitis stage was the severe form (Stage IV). Higher periodontitis severity was found to be positively associated with RT dose, plaque percentage, and CODS, suggesting factors to plan future investigation on preventive care in HNC patients. While causality cannot be inferred due to the study design, these findings could be useful in developing more effective clinical management strategies in future research.

**Trial registration:**

The study was retrospectively registered on 29/10/2024 at ClinicalTrials.gov (NCT06667362).

## Introduction

Head and neck cancer (HNC) encompasses a group of malignant tumors located above the clavicle, below the skull base, and in the anterior aspect of the neck vertebral column, including thyroid cancer, lip-oral cancer, laryngeal cancer, nasopharyngeal cancer, and other pharyngeal malignancies. These cancers represent a significant global health burden due to their high incidence and associated morbidity and mortality [1]. According to Global Cancer Statistics (2020), HNC ranks as the third most prevalent cancer worldwide, with 1,464,550 new cases and 487,993 deaths annually [2]. This accounts for 7.6% of all cancers and 4.8% of all cancer-related deaths, emphasizing the critical need for effective prevention and management strategies [3].

The primary treatment modalities for HNC include surgical resection, radiotherapy (RT), and chemotherapy, either used individually or in combination. While these interventions effectively eliminate tumors, they also result in adverse effects on surrounding structures, including soft tissues, sensory functions, and the dentition [4, 5]. Patients undergoing RT for HNC experience both acute and chronic complications affecting soft tissues and sensory functions. Acute effects include mucositis, thickened secretions, mucosal infections, pain, and sensory disturbances. Meanwhile, chronic complications often involve fibrosis, salivary gland dysfunction, neuropathic pain, and increased susceptibility to dental caries and periodontal disease [6, 7]. Additionally, cancer itself and its treatment can compromise immune function, predisposing patients to periodontal infections [8].

RT induces significant microbiological alterations, leading to oral microbiome dysbiosis characterized by a shift in microbial composition and an increase in pathogenic bacterial colonization [9]. This disruption creates an environment that exacerbates pre-existing periodontal conditions [10]. Moreover, radiation-induced xerostomia reduces saliva production, which plays a crucial role in maintaining oral health through its antimicrobial properties and buffering capacity. The resulting challenges in oral hygiene maintenance further contribute to periodontal disease progression, with potential systemic health implications [11].

Periodontal disease encompasses a range of inflammatory conditions, primarily gingivitis and periodontitis, affecting approximately 50% of the global adult population [12, 13]. Bacterial infections in periodontitis trigger a chronic inflammatory response, which gradually destroys periodontal tissues, ultimately leading to tooth loss [12]. The interrelationship between oncology and dentistry is critical for optimizing patient outcomes, particularly in the context of HNC treatment-related periodontal complications. A comprehensive understanding of how RT impacts periodontal health is essential for developing integrated care strategies that address both oncological and oral health needs [14].

Given the diverse clinical presentations of HNC, individualized treatment plans must consider factors such as tumor location, stage, and overall health to balance therapeutic efficacy with the preservation of vital functions [8]. While multiple epidemiological studies have explored the association between periodontitis disease and cancer risk, few studies have specifically examined the impact of cancer therapy on periodontal health in HNC patients [15–19].

Additionally, most of the existing studies originates from non-Middle Eastern populations, creating a significant knowledge gap regarding the oral health challenges faced by HNC patients in Egypt. To the best of our knowledge, this is the first study in an Egyptian population to evaluate the prevalence and severity of periodontal disease in HNC patients following RT and to identify associated risk factors. The findings aim to provide locally relevant evidence that can support the development of integrated dental-oncology care protocols in resource-limited setting.

## Study hypothesis

### Null hypothesis (H0)

Radiotherapy (RT) has no significant effect on the periodontal status of head and neck cancer (HNC) patients, and there are no identifiable associated risk factors affecting periodontal health post-RT.

### Alternative hypothesis (H1)

Radiotherapy (RT) may be associated with increased severity of periodontal disease in head and neck cancer (HNC) patients, and specific associated risk factors contribute to the deterioration of periodontal health post-RT.

### Subjects and methods

#### Sample size calculation

A power analysis was conducted to ensure adequate statistical power for testing the research question regarding the prevalence of periodontal disease in a sample of head and neck cancer patients from the Egyptian population. A confidence interval of 95% and a margin of error of 5% were adopted, incorporating finite population correction. The prevalence of periodontitis in end-stage renal disease patients on hemodialysis, as reported in a previous study by Abou-Bakr et al. (2022) [20]. was used as a reference. Based on these parameters, the predicted sample size (n) was determined to be 189 cases. The sample size calculation was performed using Epi Info for Windows, version 7.2 [21].

#### Study design and setting

This prospective cross-sectional study was conducted on 189 patients at Ahmed Maher Radiation Center in Cairo Governorate, Egypt, a major referral facility for cancer treatment. Data were collected through direct interviews, dental and periodontal examinations of HNC patients, and a review of medical records for relevant clinical information. The study design adhered to the guidelines of the Declaration of Helsinki.

The research proposal was reviewed and approved by the Ahmed Maher Hospital Research Ethics Committee (HAM00212). All patient data were kept confidential, and the study procedures were fully explained to each participant. Informed consent was obtained from all recruited patients before their inclusion in the study.

## The outcomes

### Primary outcomes

Periodontal Status Post-Radiotherapy:

Assessment of periodontal parameters (e.g., gingival recession, pocket depth, clinical attachment level, bleeding on probing) in HNC patients post-RT.

Frequency and severity of periodontal disease in the population studied.

### Secondary outcomes

Identification of Associated Risk Factors:

Analysis of factors such as age, gender, oral hygiene practices, nutritional status, and systemic health conditions influencing periodontal status post-RT.

## Patient selection

### Inclusion criteria

Patients included in the study were required to be over 18 years of age, of either gender, and have a minimum of six teeth present in the oral cavity. Eligible participants had completed RT to the head and neck region, with or without chemotherapy, consisting of 25–35 sessions over 6–7 weeks, with a total dose between 5000 and 7000 cGy. At least six months must have elapsed since the completion of RT [22]. The study specifically included patients diagnosed with head and neck squamous cell carcinoma, including squamous cell carcinoma of the tongue, buccal mucosa, hard palate, soft palate, and base of the tongue. In all cases, both the mandible and maxilla were within the irradiation field.

### Exclusion criteria

Patients were excluded if they had undergone major surgical interventions involving significant hard tissue resection, had psychiatric disorders, distant metastatic disease, bone-related disorders, active untreated infections, or were receiving palliative care. Pregnant patients and smokers were also excluded, as smoking is a well-established risk factor for periodontal disease [23], and its exclusion aimed to minimize potential confounders in the study sample.

### Patient’s demographic data

Data were collected through direct personal interviews conducted by the primary investigator (A.A). Patients were asked about their gender, age, presence of systemic diseases, medication intake, date of their last dental appointment, use of removable prostheses, and oral hygiene habits. Information on oral hygiene habits included whether they brushed their teeth (yes or no), the frequency of tooth brushing per day, and whether they used mouthwash.

**Fig. 1:**
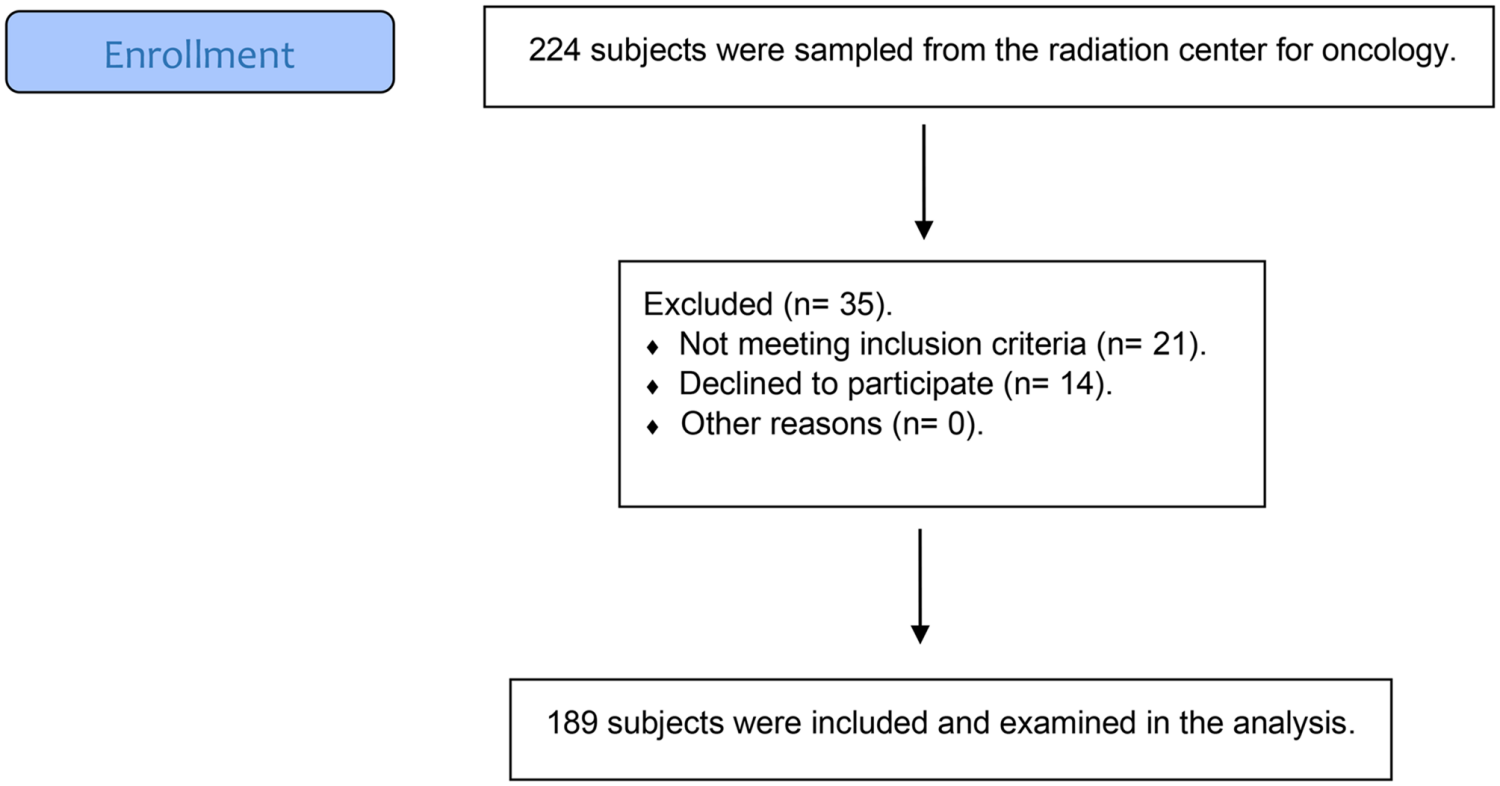
Flow diagram for HNC patients’ recruitment

Flow diagram for patients’ recruitment presented in Fig. 1.

### Hospital medical records

Cancer-related information was collected from hospital medical records. This included details on tumor location, tumor–node–metastasis (TNM) staging, histologic subtype, tumor grade differentiation, type of treatment, history of previously treated cancer, number of RT sessions, and total body mass index (BMI). The sixth edition of the TNM staging system for head and neck cancers was used to classify tumor size (T), lymph node status (N), and metastatic status (M) [24]. Tumor grade was categorized as moderately differentiated, poorly differentiated, or undifferentiated.

### Clinical periodontal examination

To reduce individual variability, all clinical measurements were assessed and recorded by a single calibrated investigator (FA), who has ten years of experience in periodontology. According to the current classification of periodontal and peri-implant disorders (2017) [25], a radiographic examination was performed on each participant to determine their periodontal status and stage of periodontitis [26]. Participants without gingivitis were defined as those with no clinical attachment loss due to periodontal disease, pocket depths of ≤ 3 mm, and bleeding on probing (BOP) scores of ≤ 10% across the entire mouth. Participants classified as having good periodontal health exhibited similar periodontal parameters; pocket depths of ≤ 3 mm and BOP scores of ≤ 10%, consistent with established criteria proposed by Trombelli et al. [27].

A Williams periodontal probe was used to assess plaque accumulation on the mesial, distal, buccal, and palatal surfaces of each tooth. The plaque index was determined by summing the values for each tooth and calculating the average. Reference values for the plaque index were used as a basis for assessment [28]:

- Plaque index 0: No plaque is in the area adjacent to the gingiva.
- Plaque index 1: There is a plaque in the form of a thin film on the gingival margin.
- Plaque index 2: There is a visible plaque in the gingival pocket and gingival margin.
- Plaque index 3: There is a dense plaque in the gingival pocket and on the gingival margin.

The plaque index (PI) was calculated by dividing the number of surfaces with plaque by the total number of available surfaces. Surfaces without soft plaque accumulation at the dento-gingival junction were not included in the analysis. Six tooth positions—mesio-buccal/facial, mid-buccal/facial, disto-buccal/facial, mesio-lingual/palatal, mid-lingual/palatal, and disto-lingual/palatal—were measured for probing depth (PD) and clinical attachment loss (CAL) using a periodontal probe, except for the third molars.

The loss of clinical attachment was quantified by measuring the distance from the base of the pocket to the cementoenamel junction (CEJ). When the probing depth (the distance from the free gingival margin to the base of the sulcus/pocket) equaled the gingival margin (the distance from the free gingival margin to the CEJ), the attachment epithelium remained intact at the CEJ, indicating no loss of clinical attachment. In other words, if the periodontal probe stopped above the CEJ while measuring the pocket depth, no clinical attachment loss was recorded [20].

Patient history was obtained to evaluate tooth loss caused by periodontitis. Patients frequently reported experiencing discomfort while eating and chewing due to excessive tooth mobility.

The bleeding on probing (BOP) score was calculated as the proportion of bleeding sites 10 s after stimulation with a standardized manual probe applied with controlled force to the bottom of the sulcus/pocket. Measurements were taken at six locations per tooth: mesio-buccal, buccal, disto-buccal, mesio-lingual, lingual, and disto-lingual.

### Case definition of gingivitis according to trombilli et al., [29] is

Probing depth ≤ 3 mm.

BOP score ≥ 10%, ≤ 30% if localized and > 30% if generalized.

Absence of clinical attachment loss.

Absence of radiographic bone loss.

Following data collection, periodontal cases were categorized according to the 2017 World Workshop classification criteria. A diagnosis of periodontitis was established based on the presence of interdental CAL detectable at ≥ 2 non-adjacent teeth, or buccal/oral CAL ≥ 3 mm with probing pocket depth > 3 mm at ≥ 2 teeth, in the absence of non-periodontal causes of attachment loss [26].

### Other clinical parameters

he Clinical Oral Dryness Score (CODS) [30–32]:

1. Mirror sticks to buccal mucosa.
2. Mirror sticks to tongue.
3. Frothy saliva.
4. No saliva pool on the floor of mouth.
5. Tongue shows loss of papillae.
6. Altered/smooth gingival architecture.
7. Glassy appearance of other oral mucosa, especially palate.
8. Tongue lobulated/fissured.
9. Active or recent (2 teeth).
10. Debris on palate (excluding under dentures).
11. A total CODS was calculated by adding the scores from the ten features. Increased severe oral dryness is indicated by a high overall score [33].

Interpretation of the CODS Score [30].

An additive score of 1–3 indicates mild dryness which may not need treatment.

An additive score of 4–6 indicates moderate dryness.

An additive score of 7–10 indicates severe dryness.

### Salivary flow rate

HNC patients were instructed to minimize facial movements and refrain from manipulating salivary flow, such as by swallowing or sucking, while seated during the saliva collection process. They were advised not to swallow for 60 s before collection to allow saliva to accumulate on the floor of the mouth. After this period, they were instructed to spit the collected saliva into a pre-weighed 50 mL vial [34]. This process was repeated four more times, resulting in a total collection time of five minutes. Throughout the procedure, patients were instructed not to consume the saliva [35]. The normal unstimulated salivary flow rate ranges between 0.3 and 0.4 mL/min. Hyposalivation is diagnosed when the unstimulated salivary flow rate falls below 0.1 mL/min [36].

### Statistical analysis

Categorical and ordinal data were presented as frequencies and percentages, while numerical data were summarized using mean, standard deviation (SD), median, and interquartile range (IQR) values. Normality was assessed through visual inspection of the distribution and the Shapiro-Wilk test, which indicated that the data were non-parametric. Associations were analyzed using the Kruskal-Wallis test, followed by Dunn’s post hoc test where applicable, while correlations were evaluated using Spearman’s rank-order correlation coefficient. Stepwise binary logistic regression models were used to explore the relationship between recession severity classes and various risk factors, with model selection based on the Akaike Information Criterion (AIC), choosing the model with the lowest AIC value.

Multinomial logistic regression models were performed to evaluate the association between periodontal disease stages (I–IV) and a set of independent variables, including RT dose, RT fraction, salivary flow rate, plaque percentage, Clinical Oral Dryness Score (CODS), and body mass index (BMI). Periodontally healthy and gingivitis patients served as the reference category. Predictor variables were selected based on clinical relevance and previous literature, and all were entered simultaneously into the models. Stepwise model selection based on the Akaike Information Criterion (AIC) was applied, and the model with the lowest AIC value was retained.

Linearity in the log odds was assessed using binned residual plots, confirming no major deviations from linearity. Multicollinearity was evaluated using the Variance Inflation Factor (VIF), with all predictors showing VIF values < 5, indicating acceptable collinearity. To evaluate model generalizability and reduce overfitting, leave-one-out cross-validation (LOOCV) was conducted prior to final analysis. Odds ratios (ORs) and 95% confidence intervals (CIs) were reported for each predictor. The significance level was set at *p* < 0.05 for all tests. Statistical analyses were conducted using R statistical software version 4.4.2 for Windows. 1

## Results

### Demographic and medical characteristics

Of the 189 participants examined, 118 (62.43%) were male and 71 (37.57%) were female, with a mean age of 51.14 ± 10.71 years. Regarding medical conditions, 13 (6.88%) participants were medically free, while 176 (93.12%) had at least one medical condition. The mean BMI was 18.77 ± 3.25. In terms of cancer family history, 136 (71.96%) participants had no family history of cancer, while 53 (28.04%) reported a positive family history. The educational levels of the participants were distributed as follows: 46 individuals (24.34%) had a low level of education, 97 individuals (51.32%) had a medium level of education, and 46 individuals (24.34%) had a high level of education. This distribution reflects a diverse range of educational backgrounds, which may influence health literacy and access to medical care, potentially impacting the management of head and neck cancer and its associated complications. These demographic characteristics are summarized in Table 1.

### Cancer characteristics

50 (26.46%) participants had a primary tumor in the larynx, 38 (20.11%) in the oropharynx, 28 (14.81%) in the nasopharynx, and 73 (38.62%) in the oral cavity. Regarding surgical intervention, 84 (44.44%) participants underwent a biopsy, while 105 (55.56%) had a total excision. The distribution of primary tumor sites was as follows: larynx in 27 (14.29%), tongue in 34 (17.99%), parotid gland in 5 (2.65%), buccal mucosa in 15 (7.94%), thyroid gland in 9 (4.76%), mandible in 15 (7.94%), nasopharynx in 19 (10.05%), floor of the mouth in 14 (7.41%), maxillary sinus in 9 (4.76%), cervical lymph node in 5 (2.65%), soft palate in 18 (9.52%), vocal cord in 9 (4.76%), lip in 5 (2.65%), and pharynx in 5 (2.65%), as shown in Table 2.

**Table 1:** Demographic data of the study participants

Parameter (*n* = 189)		Value
Gender [n (%)]	Male	118 (62.43%)
	Female	71 (37.57%)
Age	Mean ± SD	51.14 ± 10.71
	Median (IQR)	54.00 (19.00)
Medical condition [n (%)]	Free	13 (6.88%)
	Compromised	176 (93.12%)
BMI	Mean ± SD	18.77 ± 3.25
	Median (IQR)	18.30 (5.20)
Cancer family history [n (%)]	Negative	136 (71.96%)
	Positive	53 (28.04%)
Educational level [n (%)]	Low	46 (24.34%)
	Medium	97 (51.32%)
	High	46 (24.34%)

In terms of tumor staging, 75 (39.68%) participants were classified as T1, 90 (47.62%) as T2, and 24 (12.70%) as T3. For the N-stage, 55 (29.10%) participants were classified as N0, 114 (60.32%) as N1, and 20 (10.58%) as N2. Histopathology grading was as follows: grade I in 26 (13.76%), grade II in 110 (58.20%), grade III in 41 (21.69%), and grade IV in 12 (6.35%). Regarding chemoradiotherapy, 125 (66.14%) participants did not receive chemoradiotherapy, while 64 (33.86%) underwent the treatment, as presented in Table 2.

**Table 2:** Medical parameters for cancer patients

Parameter (*n* = 189)		Value
Primary tumor [n (%)]	Larynx	50 (26.46%)
	Oropharynx	38 (20.11%)
	Nasopharynx	28 (14.81%)
	Oral cavity	73 (38.62%)
Surgical intervention [n (%)]	Biopsy	84 (44.44%)
	Total excision	105 (55.56%)
Primary site [n (%)]	Larynx	27 (14.29%)
	Tongue	34 (17.99%)
	Parotid gland	5 (2.65%)
	Buccal mucosa	15 (7.94%)
	Thyroid gland	9 (4.76%)
	Mandible	15 (7.94%)
	Nasopharynx	19 (10.05%)
	Floor of the mouth	14 (7.41%)
	Maxillary sinus	9 (4.76%)
	cervical lymph node	5 (2.65%)
	Soft palate	18 (9.52%)
	Vocal cord	9 (4.76%)
	Lip	5 (2.65%)
	Pharynx	5 (2.65%)
T-stage [n (%)]	T1	75 (39.68%)
	T2	90 (47.62%)
	T3	24 (12.70%)
N-stage [n (%)]	N0	55 (29.10%)
	N1	114 (60.32%)
	N2	20 (10.58%)
Histopathology stage [n (%)]	Grade (I)	26 (13.76%)
	Grade (II)	110 (58.20%)
	Grade (III)	41 (21.69%)
	Grade (IV)	12 (6.35%)
Radiotherapy total dose	Mean ± SD	6480.42 ± 601.67
	Median (IQR)	6600.00 (1000.00)
Radiotherapy fraction	Mean ± SD	31.80 ± 3.01
	Median (IQR)	33.00 (5.00)
Chemoradiotherapy [n (%)]	No	125 (66.14%)
	Yes	64 (33.86%)

### Salivary and oral clinical data

Salivary clinical parameters including Clinical Oral Dryness Score (CODS) and salivary flow rates were presented in Table 3.

**Table 3:** Salivary clinical parameters

Parameter (*n* = 189)		Value
Clinical Oral Dryness Score (CODS)	Mean ± SD	7.61 ± 1.46
	Median (IQR)	8.00 (2.00)
Salivary flow rates/ 5 min	Mean ± SD	0.40 ± 0.31
	Median (IQR)	0.30 (0.30)

#### Prevalence of periodontal diseases in HNC patients

A total of 189 head and neck cancer (HNC) patients were examined. Among them, 183 patients (96.8%) were diagnosed with periodontal disease, while 6 patients (3.2%) were periodontally healthy. Of the 183 cases with periodontal disease, 174 (95.08%) were diagnosed with periodontitis and 9 (4.9%) with gingivitis. The distribution of periodontitis stages was as follows: stage I in 21 cases (12.07%), stage II in 30 cases (17.24%), stage III in 55 cases (31.61%), and stage IV in 68 cases (39.08%), as presented in Table 4. A detailed descriptive analysis of the clinical parameters associated with different periodontitis stages is provided in Table 5.

**Table 4:** Prevalence and severity of periodontal diseases

HNC patients (*n* = 189)				Value
HNC patients with Periodontal diseases (*n* = 189)	Yes			183 (96.8%)
	No			6 (3.17%)
Periodontal diseases 183 (96.8%)		Gingivitis		9 (4.9%)
		Periodontitis		174 (95.08%)
Periodontitis 174 (95.08%)			Stage (I)	21 (12.07%)
			Stage (II)	30 (17.24%)
			Stage (III)	55 (31.61%)
			Stage (IV)	68 (39.08%)

**Table 5:** Descriptive analysis of different periodontitis stages clinical parameters

Periodontists stage	Clinical parameter		Value
Stage (I) (*n* = 21)	**Plaque (%)**	**Mean ± SD**	18.71 ± 1.68
		**Median (IQR)**	19.00 (2.00)
	**Bleeding on probing (%)**	**Mean ± SD**	26.95 ± 4.25
		**Median (IQR)**	26.00 (5.00)
	**Deepest CAL (mm)**	**Mean ± SD**	2.00 ± 0.00
		**Median (IQR)**	2.00 (0.00)
	**Number of missing teeth**	**Mean ± SD**	0.00 ± 0.00
		**Median (IQR)**	0.00 (0.00)
Stage (II) (*n* = 30)	**Plaque (%)**	**Mean ± SD**	27.67 ± 2.87
		**Median (IQR)**	28.50 (4.75)
	**Bleeding on probing (%)**	**Mean ± SD**	37.90 ± 4.69
		**Median (IQR)**	37.50 (6.75)
	**Deepest CAL (mm)**	**Mean ± SD**	4.03 ± 0.18
		**Median (IQR)**	4.00 (0.00)
	**Number of missing teeth**	**Mean ± SD**	0.00 ± 0.00
		**Median (IQR)**	0.00 (0.00)
Stage (III) (*n* = 55)	**Plaque (%)**	**Mean ± SD**	33.04 ± 3.07
		**Median (IQR)**	33.00 (4.50)
	**Bleeding on probing (%)**	**Mean ± SD**	44.64 ± 7.21
		**Median (IQR)**	44.00 (7.50)
	**Deepest CAL (mm)**	**Mean ± SD**	5.91 ± 1.34
		**Median (IQR)**	5.00 (1.00)
	**Number of missing teeth**	**Mean ± SD**	2.45 ± 0.81
		**Median (IQR)**	2.00 (1.00)
Stage (IV) (*n* = 68)	**Plaque (%)**	**Mean ± SD**	40.32 ± 4.95
		**Median (IQR)**	39.00 (6.25)
	**Bleeding on probing (%)**	**Mean ± SD**	59.12 ± 12.67
		**Median (IQR)**	60.00 (21.25)
	**Deepest CAL (mm)**	**Mean ± SD**	8.09 ± 1.68
		**Median (IQR)**	8.00 (2.00)
	**Number of missing teeth**	**Mean ± SD**	6.85 ± 1.51
		**Median (IQR)**	6.00 (2.00)

### Correlations of different clinical parameters with periodontitis severity

**Fig. 2:**
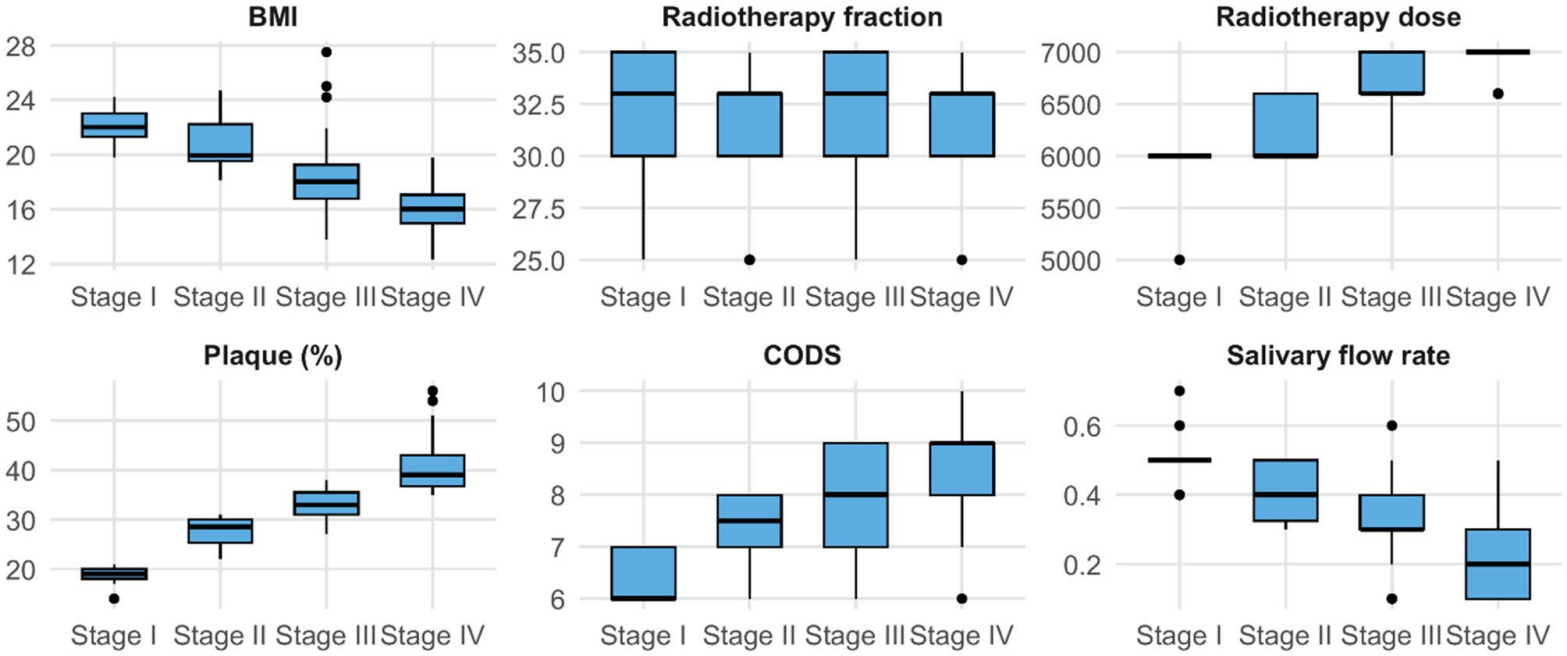
Box and whisker plots for different correlations with different periodontitis stages

No significant correlation was observed between RT fraction number and periodontitis severity (*p* = 0.837). Periodontitis stage was negatively correlated with both BMI and salivary flow rate. Positive correlations were found between periodontitis severity and RT dose, plaque percentage, and Clinical Oral Dryness Score (CODS), as presented in Table 6; Fig. 2.

**Table 6:** Correlations with periodontitis severity

Parameter	*r* (95% CI)	*p*-value
BMI	-0.770 (-0.825:-0.702)	< 0.001*
Radiotherapy fraction	-0.016 (-0.164:0.133)	0.837
Radiotherapy dose	0.777 (0.710:0.830)	< 0.001*
Plaque (%)	0.892 (0.858:0.919)	< 0.001*
CODS	0.655 (0.560:0.732)	< 0.001*
Salivary flow rate	-0.677 (-0.750:-0.588)	< 0.001*

CI Confidence interval, * significant (*p* < 0.05)

#### Regression models analysis

The overall models were statistically significant across different analyses. In the first model, χ²(1) = 128.17, *p* < 0.001, the predictor explained a substantial proportion of the variance (Nagelkerke R²), though plaque percentage was not statistically significant (*p* = 0.993) (Table 7). In the second model, χ²(8) = 30.35, *p* < 0.001, the predictors explained 26.6% of the variance (Nagelkerke R²), but none of the included variables demonstrated statistically significant associations (Table 8).

**Table 7:** Regression model prediction Stage (I) periodontitis

Term	Odds ratio	95% CI		Test statistic	*p*-value
		Lower	Upper		
Plaque	1.59E-16	0.00	∞	-0.01	0.993

CI Confidence interval

**Table 8:** Regression model prediction Stage (II) periodontitis

Term	Odds ratio	95% CI		Test statistic	*p*-value
		Lower	Upper		
Radiotherapy fraction	0.88	0.76	1.02	-1.72	0.086
Radiotherapy dose	0.998	0.9971	1.0002	-1.68	0.094
Plaque (%)	0.98	0.88	1.08	-0.48	0.632
CODS	1.65	0.74	3.82	1.21	0.227
Salivary flow rate	125.94	0.14	140126.39	1.39	0.166

CI Confidence interval

In the third model, χ²(8) = 38.94, *p* < 0.001, the predictors explained 28.1% of the variance (Nagelkerke R²), an increase in RT dose was significantly associated with higher odds of stage III periodontitis (*p* < 0.001), while other variables were not significant (Table 9), suggesting a dose-dependent trend, though causality cannot be inferred due to study design. Finally, in the fourth model, χ²(8) = 187.60, *p* < 0.001, the predictors explained 89.4% of the variance (Nagelkerke R²), and an increase in plaque percentage was significantly associated with higher odds of stage IV periodontitis (*p* < 0.001), while other variables showed no significant effects (Table 10).

**Table 9:** Regression model prediction Stage (III) periodontitis

Term	Odds ratio	95% CI		Test statistic	*p*-value
		Lower	Upper		
Radiotherapy fraction	0.94	0.81	1.08	-0.90	0.371
Radiotherapy dose	1.002	1.001	1.004	3.46	< 0.001*
Plaque (%)	0.93	0.86	1.01	-1.61	0.107
CODS	0.91	0.44	1.85	-0.25	0.801
Salivary flow rate	0.15	0.00	60.25	-0.62	0.538

CI Confidence interval, * significant (*p* < 0.05)

**Table 10:** Regression model prediction Stage (IV) periodontitis

Term	Odds ratio	95% CI		Test statistic	*p*-value
		Lower	Upper		
Radiotherapy fraction	1.07	0.78	1.45	0.43	0.665
Radiotherapy dose	1.003	0.999	1.007	1.62	0.105
Plaque (%)	2.39	1.64	4.42	3.57	< 0.001*
CODS	0.93	0.14	7.20	-0.07	0.945
Salivary flow rate	8.44E-15	0.00	1592.65	-1.10	0.271

CI Confidence interval, * significant (*p* < 0.05)

## Discussion

Maintaining good oral hygiene is essential in the multidisciplinary treatment of HNC patients, particularly when RT is used, as it increases the risk of periodontal destruction [37, 38]. High-dose RT has both direct and indirect effects on the periodontium, leading to a greater risk of periodontal attachment loss and tooth loss. Without adequate oral hygiene, the risk of extensive periodontal destruction increases among HNC patients [39]. Consequently, these patients may be more susceptible to developing periodontal disease. However, the available literature provides limited, often inconsistent, and insufficient data regarding the prevalence of periodontitis among HNC patients after RT.

This study aimed to evaluate the frequency of periodontal disease in a representative sample of HNC patients who have received RT and to identify associated risk factors. The findings support an association between RT dose and worsened periodontal health. Strong associations were observed between periodontitis severity and RT dose, plaque percentage, and CODS. The lack of significance between RT fractionation and periodontitis severity suggests that RT dose has a greater impact than the fractionation pattern. Additionally, negative correlations with BMI and salivary flow rate align with the expected adverse effects of RT on oral health.

Thus, the Alternative Hypothesis (H1) is partially confirmed, indicating that while RT dose is significantly associated with periodontal health outcomes, while the number of RT fractions alone is not a strong determinant of periodontal severity [37–39].

Regarding the demographic data, males constituted 62.43% of the study population, while females made up 37.57%. This finding aligns with an earlier study by [40], which reported a higher prevalence of HNC in males (79.2%) than in females (20.7%). Similarly, a study by Rupe et al. (2022) found that males with HNC were more prevalent (65.64%) than females (34.35%). Several descriptive epidemiological studies also suggest that men are more likely than women to develop head and neck malignancies [41–43]. Sex hormones may play a crucial role in carcinogenesis and cancer susceptibility, as estrogens appear to have protective effects in females, whereas androgens are strongly associated with a higher cancer prevalence and worse outcomes in males [44, 45].

The mean age in the current study was 51.14 ± 10.71 years, which is consistent with previous studies on HNC patients, where the mean age was reported to be over 50 years [46–49]. Additionally, 176 participants (93.12%) had compromised medical conditions, while 13 participants (6.88%) were free of any medical conditions. The link between chronic systemic inflammation and malignancy risk is well established [50, 51]. This finding aligns with prior research that has identified systemic diseases, infections, genetic disorders, and medical treatments as contributing factors to the increased risk of head and neck carcinoma [52, 53].

Regarding the Clinical Oral Dryness Score (CODS), the mean score was 7.61 ± 1.46, with a median of 8.00 (IQR 2.00), indicating severe oral dryness according to CODS score interpretation (Challacombe & Stephen, 2015). For salivary flow rates, the mean was 0.40 ± 0.31, with a median of 0.30 (IQR 0.30), which confirms hyposalivation [36]. These findings are consistent with previous research, which reported that 78.41% of HNC patients experienced hyposalivation [54]. Notably, decreased salivary flow has been identified as one of the most frequently documented late side effects of RT for HNC [6, 55–59].

Hyposalivation, or reduced salivary flow, significantly impacts periodontal tissues, contributing to the development of periodontitis [60]. Saliva plays a crucial role in maintaining a balanced oral microbiome. When its production decreases, microbial dysbiosis occurs, allowing the proliferation of pathogenic bacteria such as Porphyromonas gingivalis—a key contributor to periodontal disease [61]. Additionally, Streptococcus mutans, primarily known for its role in dental caries, can interact with other oral bacteria, further aggravating periodontal conditions [62]. This bacterial shift significantly increases the risk of gingivitis and periodontitis, particularly in individuals with hyposalivation [63].

One major consequence of hyposalivation is the reduced natural flushing action of saliva, leading to plaque and calculus accumulation, which directly contributes to periodontal disease. As a result, hyposalivation has profound implications not only for oral health but also for overall well-being [63]. The dysbiosis-induced inflammatory response triggers an exaggerated immune reaction, leading to destruction of periodontal tissues and disease progression [64, 65].

Furthermore, dehydration of gingival tissues due to insufficient saliva weakens the gum tissues, making them fragile, inflamed, or ulcerated, thereby increasing susceptibility to infections [66]. Lastly, saliva contains essential growth factors necessary for wound healing and tissue regeneration. The absence of these factors in individuals with hyposalivation slows down periodontal healing and compromises tissue regeneration [67]. These interconnected effects emphasize the critical role of saliva in maintaining oral homeostasis, supporting immune defense, and preserving periodontal health. Therefore, addressing hyposalivation is essential for effectively preventing and managing periodontal disease.

In the current study, 183 (96.8%) HNC patients were found to have periodontal disease. Among them, 174 (95.08%) were diagnosed with periodontitis, while 9 (4.9%) were diagnosed with gingivitis. The distribution of periodontitis different stages was as follows: Stage I was found in 21 (12.07%) cases, Stage II in 30 (17.24%), Stage III in 55 (31.61%), and Stage IV in 68 (39.08%).

The diverse study designs and diagnostic criteria in previous studies mostly limits direct comparisons with our results. However, some research has yielded comparable findings. Komlós et al. (2021) found that most HNC patients had stage IV periodontitis (72.1%), followed by stage III (14%) [16]. Similarly, Rupe et al. (2022) reported that stage I periodontitis was 14%, stage II was 14.7%, stage III was 28%, with the most prevalent being stage IV (43.3%). Additionally, they found that severe periodontitis (stages III and IV) was diagnosed only in subjects aged > 40 years, and 93.5% of periodontitis patienst were older than 49 years, with a total periodontitis prevalence of 76.9%. Multiple logistic regression analysis in the same study by Rupe et al. (2022) showed that age (in decades) was a risk factor for periodontitis (stage I and II: OR 1.73, 95% CI = 1.15–2.61; stage III and IV: OR 3.30, 95% CI = 2.17–5.00; *p* < 0.05) [68].

Although previous studies used different classification systems, our results align with those of Epstein et al. (1998), who concluded that patients receiving irradiation therapy for cancer experience tooth loss and increased periodontal attachment loss in teeth located within high-dose radiated areas [69].

Bonan et al. (2006) reported a 93% prevalence of moderate or severe periodontitis [70], while a study by Critchlow et al. (2014) found a slightly lower prevalence at 80% [71]. Similarly, Moraes et al. (2016) observed that 80% of patients with oral and oropharyngeal SCC had generalized chronic periodontitis, with nearly all cases classified as severe [72].

Additionally, Sharma et al. (2020) found that all periodontal parameters significantly worsened after RT in HNC patients. A 3–4 mm CAL (moderate grade) was recorded in 56% of the studied participants. The mean ± standard deviation (SD) of CAL before and after RT therapy was 2.48 ± 1.08 and 3.66 ± 1.27, respectively. The statistical analysis of pre-RT and post-RT readings indicated a very significant difference (*P* < 0.001), highlighting the damaging effects of RT on the periodontium [22].

Only a few studies have adopted the 2017 classification criteria for periodontitis [73, 74]. A recent review found that studies exploring a potential link between periodontitis and HNC vary significantly in methodology and often have poor research design [73, 74]. Specifically, only one study conducted a clinical evaluation with truly appropriate measures such as PPD (probing pocket depth) and CAL [72], while most studies [75–77] lacked standardized diagnostic criteria for periodontitis.

The 2017 classification of periodontitis aims to provide a more detailed and accurate categorization of periodontal disease, addressing methodological issues observed in epidemiological studies [26] This system was used in our study to classify all periodontitis cases. Using prior classification may have underestimated disease severity in patients with advanced oral deterioration. Although the lack of comparability limits direct interpretation, our results strongly suggest a high periodontitis prevalence in HNC patients. However, causality cannot be inferred due to the study design.

In the current study, the mean plaque accumulation (%) was found to be 18.71 ± 1.68 in Stage I periodontitis, 27.67 ± 2.87 in Stage II, 33.04 ± 3.07 in Stage III, and 40.32 ± 4.95 in Stage IV, with the highest values observed in Stage IV. Similarly, Marciani et al. (1992) reported a high plaque index in irradiated cancer patients [78]. De Moraes et al. (2016) also reported a plaque index of 21.7% in HNC patients [72]. Additionally, Komlós et al. (2021) found that the mean plaque index (%) in oral cancer patients was 2.6 ± 0.8, significantly higher than in the healthy control group (1.6 ± 0.9) [16].

In this study, increased periodontitis severity was significantly associated with higher RT dose, greater plaque percentage, and higher CODS scores. Additionally, a strong negative correlation was observed between periodontitis severity and salivary flow rate. These findings of the present study suggest that the side-effects of radiation therapy, such as decreased salivary function, mucosal changes, and poor oral hygiene, would influence the deterioration of periodontal condition during or shortly after RT treatment. Although baseline pretreatment periodontal condition of the patients was not evaluated, these associations following treatment emphasize the need for regular early dental prophylaxis and monitoring of periodontal health in head and neck cancer patients who receive RT.

RT has a dose-dependent effect on periodontal health and is associated with worsening periodontal conditions after treatment initiation [79]. Given that pre-existing periodontitis is common in adults, it is likely to worsen once cancer treatment begins [6]. Furthermore, RT disrupts blood flow, leading to tissue hypoxia and increasing vulnerability to periodontal disease [80]. While we do not claim direct causality due to the absence of baseline data, these findings are consistent with the biological pathways and clinical patterns reported in the literature [81].

Radiation-induced vascular damage affects periodontal blood vessels, leading to the widening of the periodontal ligament space and destruction of bony trabeculae. This may result in an increased risk of periodontal disease, impaired healing, and a diminished capacity for bone remodeling and repair [69]. Llory et al. (1972) demonstrated a radiation-induced downshift of periopathogens [82], while Schwarze et al. (1999) found a greater number of periodontal pockets in radiated patients [83]. Studies by Epstein et al. (2001) demonstrated that the direct and indirect effects of high-dose RT on the periodontium result in increased attachment loss [84]. Similarly, Marx et al. (1987) reported that irradiation doses exceeding 7000 rads significantly increase the risk and severity of osteoradionecrosis [85]. Additionally, Marciani et al. (1992) indicated that smaller irradiation doses administered at higher dose rates may be more injurious than higher doses given at lower dose rates [39]. Supporting this, Marques and Dib (2004) stated that RT contributes to the progression of attachment loss to varying degrees [86].

Radiation-induced obliterative endarteritis leads to soft tissue ischemia and fibrosis, while exposed bone becomes hypovascular and hypoxic. High doses of RT can severely impact the periodontium, causing loss of cellularity in the periodontal membrane, rupture of Sharpey’s fibers, fiber thickening, and disorientation, as well as widening of the periodontal gap [22]. Both Marciani et al. (1992) and Leung et al. (1998) reported that radiation-induced hyposalivation, combined with increased plaque accumulation and shifts in oral microflora, heightens the risk of periodontal infection [78, 87]. Furthermore, poor oral hygiene often due to a fragile and irritated oral mucosa following RT contributes to periodontal attachment loss [88]. After RT completion, approximately 70% of patients experience an increased periodontal attachment loss when their oral cavity falls within the radiation field [88, 89]. Therefore, dental assessment and risk mitigation before RT is essential.

The relationship between hyposalivation and periodontitis has been extensively studied, with numerous findings identifying hyposalivation as a major risk factor for periodontal disease [60, 90, 91]. Reduced salivary flow, along with inadequate oral hygiene during and after RT, is a key contributor to increased plaque accumulation. Other factors that impair oral hygiene maintenance include trismus, anxiety, decreased physical and mental motivation, and xerostomia. In post-RT patients, maintaining plaque control is essential to prevent bacterial colonization of the gingival crevice, which could further exacerbate periodontal disease [92].

Given these interactions, customized oral hygiene instructions for patients undergoing radiation therapy are essential. Effective plaque removal methods require special attention, as weakened immune systems can worsen periodontal disease. The inclusion of remineralizing agents and antibacterial mouthwashes has been shown to provide benefits [8].

The current study also showed a strong negative correlation between periodontal disease severity and BMI in HNC patients. This finding contrasts with previous studies, which suggested that increased BMI may be a potential risk factor for periodontitis [93–97]. However, it is important to note that none of these studies focused on HNC patients, making direct comparisons inapplicable. Most research indicates that HNC is associated with lower BMI compared to normal or higher BMI populations [98–100]. A meta-analysis by Gaudet et al., which analyzed data from 17 case-control studies (12,716 cases and 17,439 controls), found that a low BMI was linked to an increased risk of HNC, independent of smoking and alcohol consumption [101].

Maintaining good oral hygiene before, during, and after radiation therapy plays an important role in preserving periodontal health in HNC population. Patients should receive expert dental care to improve oral hygiene status, prevent radiation-related complications, and reduce oral and periodontal side effects of RT. Continuous oral hygiene reinforcement is crucial throughout the course of radiation therapy to minimize complications. Since improved dental care may lower post-radiation complications affecting the periodontium, periodontists should be integrated into multidisciplinary oncology teams to provide specialized care that may enhance long-term oral health outcomes.

### Limitations

Due to the cross-sectional study design, periodontal assessments were evaluated only once post- radiotherapy, without baseline records, which limits the ability to determine changes over ime or establish causality. Moreover, the lack of a healthy non-irradiated control group makes it difficult to compare HNC patients’ periodontal health with that of general population, and interferes with the contextual interpretation of prevalence rates. Furthermore, some significant periodontal parameters that reflect advanced periodontal disease, such as furcation involvement and intraosseous defects, were not assessed in the current study, which could underestimate the severity of the periodontal disease. Clinical examination was performed during the recall visits of patients and presence of common post-radiotherapy complications, including oral dryness and trismus could have impacted the precision of periodontal measurements. Although, several possible confounders were adjusted (including smoking habits), there may be residual confounding owing to unmeasured variables like alcohol use, socioeconomic status, and a baseline oral hygiene habit. Additionally, two of the regression models showed limited explanatory power which may indicate there are more unobserved factors influencing periodontal outcomes. Finally, the generalizability to other HNC population is restricted (have a limited body of evidence as the genetic background, environmental exposures, healthcare access, lifestyle behaviors, oral hygiene practices differs between different populations).

### Future directions

Additional multicenter studies with larger sample size and diverse patient populations are necessary for the generalizability of these findings. Prospective longitudinal study design and baseline periodontal evaluations should be taken into consideration to improve better isolation of the individual influence of RT relative to radiation exposure on the progression of periodontal disease and to separate it from intrinsic disease relevance to the cancer or preexisting oral conditions.

## Conclusions

The prevalence of periodontitis among HNC patients following RT was 95.08%, reflecting a notably high burden. The most prevalent periodontitis stage was the severe form (Stage IV). Higher periodontitis severity was found to be positively associated with RT dose, plaque percentage, and CODS, suggesting factors to plan future investigation on preventive care in HNC patients. Before beginning radiotherapy, it is strongly recommended to provide a complete dental examination. Early detection and management of oral complications before and during cancer treatment with radiotherapy can decrease treatment-induced morbidity and ensure a better quality of life for HNC patients. While causality cannot be inferred due to the cross-sectional design, these findings could be useful in developing more effective clinical management strategies in future research.

## Data Availability

Research data supporting this publication is available from the corresponding author upon request.

## Footnotes

1 R Core Team (2024). R: A language and environment for statistical computing. R Foundation for Statistical Computing, Vienna, Austria. URL https://www.R-project.org/.

## References

[1] Zhou T, Huang W, Wang X, Zhang J, Zhou E, Tu Y et al. Global burden of head and neck cancers from 1990 to 2019. *iScience* 2024, 27(3):109282 10.1016/j.isci.2024.109282 10.1016/j.isci.2024.109282 PMC10918270 38455975

[2] Sung H, Ferlay J, Siegel RL, Laversanne M, Soerjomataram I, Jemal A, Bray F. Global Cancer statistics 2020: GLOBOCAN estimates of incidence and mortality worldwide for 36 cancers in 185 countries. CA Cancer J Clin. 2021;71(3):209–49. 10.3322/caac.21660. 33538338 10.3322/caac.21660

[3] Johnson DE, Burtness B, Leemans CR, Lui VWY, Bauman JE, Grandis JR. Head and neck squamous cell carcinoma. Nat Rev Dis Primers. 2020;6(1):92. 10.1038/s41572-020-00224-3. 33243986 10.1038/s41572-020-00224-3 PMC7944998

[4] Hancock PJ, Epstein JB, Sadler GR. Oral and dental management related to radiation therapy for head and neck cancer. J Can Dent Assoc. 2003;69(9):585–90. 14653934

[5] Anderson G, Ebadi M, Vo K, Novak J, Govindarajan A, Amini A. An updated review on head and neck Cancer treatment with radiation therapy. Cancers (Basel). 2021;13(19). 10.3390/cancers13194912. 10.3390/cancers13194912 PMC8508236 34638398

[6] Sroussi HY, Epstein JB, Bensadoun RJ, Saunders DP, Lalla RV, Migliorati CA, Heaivilin N, Zumsteg ZS. Common oral complications of head and neck cancer radiation therapy: mucositis, infections, saliva change, fibrosis, sensory dysfunctions, dental caries, periodontal disease, and osteoradionecrosis. Cancer Med. 2017;6(12):2918–31. 10.1002/cam4.1221. 29071801 10.1002/cam4.1221 PMC5727249

[7] Ibrahim SS, Hassanein FEA, Zaky HW, Gamal H. Clinical and biochemical assessment of the effect of glutamine in management of radiation induced oral mucositis in patients with head and neck cancer: randomized controlled clinical trial. J Stomatology Oral Maxillofacial Surg. 2024;125(3, Supplement):101827. 10.1016/j.jormas.2024.101827. 10.1016/j.jormas.2024.101827 38493953

[8] Khan N, Pasha Z. QC No. OAR-23-117662 (Q) Revised. 2023.

[9] Al-Nawas B, Grotz KA. Prospective study of the long term change of the oral flora after radiation therapy. Support Care Cancer. 2006;14(3):291–6. 10.1007/s00520-005-0895-3. 16341728 10.1007/s00520-005-0895-3

[10] Napenas JJ, Brennan MT, Bahrani-Mougeot FK, Fox PC, Lockhart PB. Relationship between mucositis and changes in oral microflora during cancer chemotherapy. oral surg Oral Med oral Pathol oral radiol endod. 2007;103(1):48–59. 10.1016/j.tripleo.2005.12.016. 17178494 10.1016/j.tripleo.2005.12.016

[11] Pushalkar S, Ji X, Li Y, Estilo C, Yegnanarayana R, Singh B, Li X, Saxena D. Comparison of oral microbiota in tumor and non-tumor tissues of patients with oral squamous cell carcinoma. BMC Microbiol. 2012;12:144. 10.1186/1471-2180-12-144. 22817758 10.1186/1471-2180-12-144 PMC3507910

[12] Martinez-Garcia M, Hernandez-Lemus E. Periodontal inflammation and systemic diseases: an overview. Front Physiol. 2021;12:709438. 10.3389/fphys.2021.709438. 34776994 10.3389/fphys.2021.709438 PMC8578868

[13] Ras AA, Kheir El Din NH, Talaat AM, Hussein RR, Khalil E. mucocutaneous changes in end-Stage renal disease under regular hemodialysis - A cross-sectional study. Indian J Dent Res. 2023;34(2):130–5. 10.4103/ijdr.IJDR_802_20. 37787198 10.4103/ijdr.IJDR_802_20

[14] Thanvi J, Bumb D. Impact of dental considerations on the quality of life of oral cancer patients. Indian J med paediatr oncol. 2014;35(1):66–70. 10.4103/0971-5851.133724. 25006287 10.4103/0971-5851.133724 PMC4080666

[15] Nwizu N, Wactawski-Wende J, Genco RJ. Periodontal disease and cancer: epidemiologic studies and possible mechanisms. Periodontol 2000. 2020;83(1):213–33. 10.1111/prd.12329. 32385885 10.1111/prd.12329 PMC7328760

[16] Komlos G, Csurgay K, Horvath F, Pelyhe L, Nemeth Z. Periodontitis as a risk for oral cancer: a case-control study. BMC oral health. 2021;21(1):640. 10.1186/s12903-021-01998-y. 34911520 10.1186/s12903-021-01998-y PMC8672540

[17] Chen SH, Chen JF, Hung YT, Hsu TJ, Chiu CC, Kuo SJ. Exploring the relationship between periodontitis, Anti-Periodontitis therapy, and Extra-Oral Cancer risk: findings from a nationwide Population-Based study. Biomedicines. 2023;11(7). 10.3390/biomedicines11071949. 10.3390/biomedicines11071949 PMC10377021 37509588

[18] Higham J, Scannapieco FA. Epidemiological associations between periodontitis and cancer. periodontol 2000. 2024;96(1):74–82. 10.1111/prd.12599. 39302022 10.1111/prd.12599

[19] Ma Y, Tuerxun N, Maimaitili G. Periodontitis and the risk of oral cancer: a meta-analysis of case-control studies. Acta Odontol Scand. 2024;83. 10.2340/aos.v83.40478. 10.2340/aos.v83.40478 PMC11302657 38742908

[20] Abou-Bakr A, Hussein RR, Khalil E, Ahmed E. The frequency of periodontitis in end-stage renal disease on Hemodialysis in a sample of Egyptian population: multi-center clinical cross-sectional study. BMC Oral Health. 2022;22(1):1. 10.1186/s12903-021-02032-x. 34980089 10.1186/s12903-021-02032-x PMC8725326

[21] Dean AG, Dean JA, Burton AH, Dicker RC, Coulombier D, Brendel KAet al: Epi Info: a word-processing, database, and statistics program for public health on IBM-compatible microcomputers [computer file] / program design by Andrew G. Dean…et al.]; programming by Jeffrey A. Dean… [et al.]; manual by Andrew G. Dean. In., Version 6.03 July 1995 edn: Atlanta, Georgia: Centers for Disease Control and Prevention;1995.

[22] Sharma G, Kandwal A, Gupta M, Ahmad M, Lal A. Assessment of periodontal changes in patients undergoing radiotherapy for Head-and-Neck malignancy. J Radiation Cancer Res 2020, 11(1).

[23] Petersen PE, Ogawa H. The global burden of periodontal disease: towards integration with chronic disease prevention and control. Periodontol 2000. 2012;60(1):15–39. 10.1111/j.1600-0757.2011.00425.x. 22909104 10.1111/j.1600-0757.2011.00425.x

[24] Patel SG, Shah JP. TNM staging of cancers of the head and neck: striving for uniformity among diversity. Cancer J Clin. 2005;55(4):242–58. 10.3322/canjclin.55.4.242. 10.3322/canjclin.55.4.242 16020425

[25] Caton JG, Armitage G, Berglundh T, Chapple ILC, Jepsen S, Kornman KS, et al. A new classification scheme for periodontal and peri-implant diseases and conditions– introduction and key changes from the 1999 classification. j clin periodontol. 2018;45(S20):S1–8. 10.1111/jcpe.12935. 29926489 10.1111/jcpe.12935

[26] Papapanou PN, Sanz M, Buduneli N, Dietrich T, Feres M, Fine DH, et al. Periodontitis: consensus report of workgroup 2 of the 2017 world workshop on the classification of periodontal and peri-implant diseases and conditions. j periodontol. 2018;89(Suppl 1):S173–82. 10.1002/JPER.17-0721. 29926951 10.1002/JPER.17-0721

[27] Trombelli L, Farina R, Silva CO, Tatakis DN. Plaque-induced gingivitis: case definition and diagnostic considerations. J Periodontol. 2018;89(Suppl 1):S46–73. 10.1002/JPER.17-0576. 29926936 10.1002/JPER.17-0576

[28] Silness J, Loe H. periodontal disease in pregnancy. II. correlation between oral hygiene and periodontal condtion. Acta Odontol Scand. 1964;22:121–35. 10.3109/00016356408993968. 14158464 10.3109/00016356408993968

[29] Trombelli L, Farina R, Silva CO, Tatakis DN. Plaque-induced gingivitis: case definition and diagnostic considerations. J Clin Periodontol. 2018;45(Suppl):S44–67. 10.1111/jcpe.12939. 29926492 10.1111/jcpe.12939

[30] Challacombe SJ, Osailan SM, Proctor GB. Clinical Scoring Scales for Assessment of Dry Mouth. In: Dry Mouth: A Clinical guide on causes, effects and treatments. edn. edited by carpenter g. berlin, heidelberg: springer berlin heidelberg; 2015: 119–132 10.1007/978-3-642-55154-3_8

[31] Pramanik R, Shirlaw P, Proctor G, Challacombe S. Clinical assessment of oral dryness: development of a scoring system related to salivary flow and mucosal wetness. oral surg oral med oral pathol oral radiol. 2012;114:597–603. 10.1016/j.oooo.2012.05.009. 22959491 10.1016/j.oooo.2012.05.009

[32] Frigaard J, Hynne H, Jensen JL. Development and proposal of a novel scoring system to classify dry mouth severity. Appl Sci. 2023;13(21):11758.

[33] Jager DHJ, Bots CP, Forouzanfar T, Brand HS. Clinical oral dryness score: evaluation of a new screening method for oral dryness. Odontology. 2018;106(4):439–44. 10.1007/s10266-018-0339-4. 29356914 10.1007/s10266-018-0339-4 PMC6153998

[34] Chambers MS, Tomsett KL, Artopoulou II, Garden AS, El-Naggar AK, Martin JW, Keene HJ. Salivary flow rates measured during radiation therapy in head and neck cancer patients: a pilot study assessing salivary sediment formation. J Prosthet Dent. 2008;100(2):142–6. 10.1016/S0022-3913(08)60160-2. 18672129 10.1016/S0022-3913(08)60160-2

[35] Navazesh M, Kumar SKS. Measuring salivary flow. J Am Dent Assoc. 2008;139:S35–40. 10.14219/jada.archive.2008.0353 18460678

[36] Pedersen AM, Bardow A, Jensen SB, Nauntofte B. Saliva and Gastrointestinal functions of taste, mastication, swallowing and digestion. Oral Dis. 2002;8(3):117–29. 10.1034/j.1601-0825.2002.02851.x. 12108756 10.1034/j.1601-0825.2002.02851.x

[37] Berrone M, Lajolo C, De Corso E, Settimi S, Rupe C, Crosetti E, Succo G. Cooperation between ENT surgeon and dentist in head and neck oncology. acta otorhinolaryngol ital. 2021;41(Suppl 1):S124–37. 10.14639/0392-100X-suppl.1-41-2021-13. 34060528 10.14639/0392-100X-suppl.1-41-2021-13 PMC8172104

[38] Lajolo C, Rupe C, Gioco G, Troiano G, Patini R, Petruzzi M, Micciche F, Giuliani M. Osteoradionecrosis of the jaws due to teeth extractions during and after radiotherapy: A systematic review. Cancers (Basel). 2021;13(22). 10.3390/cancers13225798. 10.3390/cancers13225798 PMC8616343 34830954

[39] Kassim N, Sirajuddin S, Biswas S, Rafiuddin S, Apine A. Iatrogenic damage to the periodontium caused by radiation and radiotherapy. Open Dent J. 2015;9:182–6. 10.2174/1874210601509010182. 26312083 10.2174/1874210601509010182 PMC4541411

[40] Park JO, Nam IC, Kim CS, Park SJ, Lee DH, Kim HB, Han KD, Joo YH. Sex differences in the prevalence of head and neck cancers: A 10-Year Follow-Up study of 10 million healthy people. cancers (Basel). 2022;14(10). 10.3390/cancers14102521. 10.3390/cancers14102521 PMC9139445 35626129

[41] Bray F, Ferlay J, Soerjomataram I, Siegel RL, Torre LA, Jemal A. Global cancer statistics 2018: Globocan estimates of incidence and mortality worldwide for 36 cancers in 185 countries. CA Cancer J Clin. 2018;68(6):394–424. 10.3322/caac.21492. 30207593 10.3322/caac.21492

[42] Ward EM, Sherman RL, Henley SJ, Jemal A, Siegel DA, Feuer EJ et al. Annual report to the nation on the status of cancer, featuring cancer in men and women age 20–49 years. j natl cancer inst 2019, 111(12):1279–1297 10.1093/jnci/djz106 10.1093/jnci/djz106 PMC6910179 31145458

[43] Dong M, Cioffi G, Wang J, Waite KA, Ostrom QT, Kruchko C, et al. Sex differences in Cancer incidence and survival: A Pan-Cancer analysis. Cancer Epidemiol Biomarkers Prev. 2020;29(7):1389–97. 10.1158/1055-9965.EPI-20-0036. 32349967 10.1158/1055-9965.EPI-20-0036

[44] Hsu JW, Hsu I, Xu D, Miyamoto H, Liang L, Wu XR, Shyr CR, Chang C. Decreased tumorigenesis and mortality from bladder cancer in mice lacking urothelial androgen receptor. Am J Pathol. 2013;182(5):1811–20. 10.1016/j.ajpath.2013.01.018. 23499463 10.1016/j.ajpath.2013.01.018 PMC3644728

[45] Clocchiatti A, Cora E, Zhang Y, Dotto GP. Sexual dimorphism in cancer. Nat Rev Cancer. 2016;16(5):330–9. 10.1038/nrc.2016.30. 27079803 10.1038/nrc.2016.30

[46] Cruvinel P, Pereira R, Fressatti A, Motta B, Motta A, Oliveira H, Ricz H, Macedo L, Tirapelli C. cross-sectional study of 1-to-5- year head and neck cancer survivors. brazilian j oncol. 2022;18. 10.5935/2526-8732.20220316.

[47] Dhull AK, Atri R, Dhankhar R, Chauhan AK, Kaushal V. Major risk factors in head and neck cancer: A retrospective analysis of 12-year experiences. world j oncol. 2018;9(3):80–4. 10.14740/wjon1104w. 29988794 10.14740/wjon1104w PMC6031231

[48] Hamilton SN, Mahdavi S, Martinez IS, Afghari N, Howard F, Tran E, Goddard K. A cross-sectional assessment of long-term effects in adolescent and young adult head and neck cancer survivors treated with radiotherapy. j cancer surviv. 2022;16(5):1117–26. 10.1007/s11764-021-01103-w. 34542836 10.1007/s11764-021-01103-w

[49] Kouka M, Hermanns I, Schlattmann P, Guntinas-Lichius O. The association between patient’s age and head and neck cancer treatment decision-a population-based diagnoses-related group-based nationwide study in germany. cancers (basel). 2023;15(6). 10.3390/cancers15061780. 10.3390/cancers15061780 PMC10046600 36980666

[50] Karin M, Lawrence T, Nizet V. Innate immunity gone awry: linking microbial infections to chronic inflammation and cancer. Cell. 2006;124(4):823–35. 10.1016/j.cell.2006.02.016. 16497591 10.1016/j.cell.2006.02.016

[51] van Kempen LC, de Visser KE, Coussens LM. Inflammation, proteases and cancer. Eur J Cancer. 2006;42(6):728–34. 10.1016/j.ejca.2006.01.004. 16524717 10.1016/j.ejca.2006.01.004

[52] Shephard MK, Hullah EA. Systemic diseases with an increased risk of oral squamous cell carcinoma. In., edn.: Springer Singapore; 2019. pp. 119–58. 10.1007/978-981-13-2931-9_7.

[53] Murphy BA, Wulff-Burchfield E, Ghiam M, Bond SM, Deng J. Chronic systemic symptoms in head and neck Cancer patients. jnci monogr. 2019;2019(53):lgz004. 10.1093/jncimonographs/lgz004. 10.1093/jncimonographs/lgz004 31425597

[54] Schulz RE, Bonzanini LIL, Ortigara GB, Soldera EB, Danesi CC, Antoniazzi RP, Ferrazzo KL. Prevalence of hyposalivation and associated factors in survivors of head and neck cancer treated with radiotherapy. J Appl Oral Sci. 2021;29:e20200854. 10.1590/1678-7757-2020-0854. 33886946 10.1590/1678-7757-2020-0854 PMC8075291

[55] Chambers MS, Garden AS, Kies MS, Martin JW. Radiation-induced Xerostomia in patients with head and neck cancer: pathogenesis, impact on quality of life, and management. Head Neck. 2004;26(9):796–807. 10.1002/hed.20045. 15350026 10.1002/hed.20045

[56] Blanco AI, Chao KS, El Naqa I, Franklin GE, Zakarian K, Vicic M, Deasy JO. Dose-volume modeling of salivary function in patients with head-and-neck cancer receiving radiotherapy. Int J Radiat Oncol Biol Phys. 2005;62(4):1055–69. 10.1016/j.ijrobp.2004.12.076. 15990009 10.1016/j.ijrobp.2004.12.076

[57] Jensen SB, Vissink A, Limesand KH, Reyland ME. Salivary Gland Hypofunction and Xerostomia in Head and Neck Radiation Patients. *J Natl Cancer Inst Monogr* 2019, 2019(53) 10.1093/jncimonographs/lgz016 10.1093/jncimonographs/lgz016 31425600

[58] Jensen SB, Pedersen AM, Vissink A, Andersen E, Brown CG, Davies AN, et al. A systematic review of salivary gland hypofunction and Xerostomia induced by cancer therapies: management strategies and economic impact. Support Care Cancer. 2010;18(8):1061–79. 10.1007/s00520-010-0837-6. 20333412 10.1007/s00520-010-0837-6

[59] Likhterov I, Ru M, Ganz C, Urken ML, Chai R, Okay D, et al. objective and subjective hyposalivation after treatment for head and neck cancer: long-term outcomes. laryngoscope. 2018;128(12):2732–9. 10.1002/lary.27224. 30325025 10.1002/lary.27224 PMC6309704

[60] Kapourani A, Kontogiannopoulos KN, Manioudaki AE, Poulopoulos AK, Tsalikis L, Assimopoulou AN, Barmpalexis P. A review on xerostomia and its various management strategies: the role of advanced polymeric materials in the treatment approaches. polym (Basel). 2022;14(5). 10.3390/polym14050850. 10.3390/polym14050850 PMC8912296 35267672

[61] How KY, Song KP, Chan KG. Porphyromonas gingivalis: an overview of periodontopathic pathogen below the gum line. front microbiol. 2016;7:53. 10.3389/fmicb.2016.00053. 26903954 10.3389/fmicb.2016.00053 PMC4746253

[62] Daep Carlo A, Lamont Richard J, Demuth Donald R. Interaction of Porphyromonas gingivalis with oral Streptococci requires a motif that resembles the eukaryotic nuclear receptor box Protein-Protein interaction domain. Infect Immun. 2008;76(7):3273–80. 10.1128/iai.00366-08. 18474648 10.1128/IAI.00366-08 PMC2446731

[63] Lynge Pedersen AM, Belstrøm D. The role of natural salivary defences in maintaining a healthy oral microbiota. J Dent. 2019;80:S3–12. 10.1016/j.jdent.2018.08.010. 30696553 10.1016/j.jdent.2018.08.010

[64] Hahnel S, Behr M, Handel G, Bürgers R. Saliva substitutes for the treatment of radiation-induced xerostomia—a review. Support Care Cancer. 2009;17(11):1331–43. 10.1007/s00520-009-0671-x. 19495809 10.1007/s00520-009-0671-x

[65] Suárez LJ, Garzón H, Arboleda S, Rodríguez A. Oral dysbiosis and autoimmunity: from local periodontal responses to an imbalanced systemic immunity. A review. front immunol 2020, 11. 10.3389/fimmu.2020.591255 PMC7754713 33363538

[66] Dawes C. Salivary flow patterns and the health of hard and soft oral tissues. J Am Dent Association. 2008;139:S18–24. 10.14219/jada.archive.2008.0351. 10.14219/jada.archive.2008.0351 18460676

[67] Sardellitti L, Bortone A, Filigheddu E, Serralutzu F, Milia EP. Xerostomia: from Pharmacological treatments to traditional Medicine-An overview on the possible clinical management and prevention using systemic approaches. Curr Oncol. 2023;30(5):4412–26. 10.3390/curroncol30050336. 37232794 10.3390/curroncol30050336 PMC10216964

[68] Rupe C, Basco A, Schiavelli A, Cassano A, Micciche’ F, Galli J, Cordaro M, Lajolo C. Oral health status in patients with head and neck Cancer before radiotherapy: baseline description of an observational prospective study. Cancers. 2022;14(6):1411. 35326564 10.3390/cancers14061411 PMC8945997

[69] Epstein JB, Lunn R, Le N, Stevenson-Moore P. Periodontal attachment loss in patients after head and neck radiation therapy. oral surg oral med oral pathol oral radiol endod. 1998;86(6):673–7. 10.1016/s1079-2104(98)90202-5. 9868723 10.1016/s1079-2104(98)90202-5

[70] Bonan PR, Lopes MA, Pires FR, Almeida OP. Dental management of low socioeconomic level patients before radiotherapy of the head and neck with special emphasis on the prevention of osteoradionecrosis. Braz Dent J. 2006;17(4):336–42. 10.1590/s0103-64402006000400013. 17262149 10.1590/s0103-64402006000400013

[71] Critchlow SB, Morgan C, Leung T. The oral health status of pre-treatment head and neck cancer patients. Br Dent J. 2014;216(1):E1. 10.1038/sj.bdj.2013.1246. 24413141 10.1038/sj.bdj.2013.1246

[72] Moraes RC, Dias FL, Figueredo CM, Fischer RG. Association between chronic periodontitis and oral/oropharyngeal Cancer. Braz Dent J. 2016;27(3):261–6. 10.1590/0103-6440201600754. 27224557 10.1590/0103-6440201600754

[73] Graetz C, Mann L, Krois J, Salzer S, Kahl M, Springer C, Schwendicke F. Comparison of periodontitis patients’ classification in the 2018 versus 1999 classification. J clin periodontol. 2019;46(9):908–17. 10.1111/jcpe.13157. 31152600 10.1111/jcpe.13157

[74] Ravida A, Qazi M, Troiano G, Saleh MHA, Greenwell H, Kornman K, Wang HL. Using periodontal staging and grading system as a prognostic factor for future tooth loss: A long-term retrospective study. J periodontol. 2020;91(4):454–61. 10.1002/JPER.19-0390. 31502244 10.1002/JPER.19-0390

[75] Guha N, Boffetta P, Wunsch Filho V, Eluf Neto J, Shangina O, Zaridze D, et al. Oral health and risk of squamous cell carcinoma of the head and neck and esophagus: results of two multicentric case-control studies. Am J Epidemiol. 2007;166(10):1159–73. 10.1093/aje/kwm193. 17761691 10.1093/aje/kwm193

[76] Saira, Ahmed R, Malik S, Khan MF, Khattak MR. Epidemiological and clinical correlates of oral squamous cell carcinoma in patients from north-west Pakistan. J pak med assoc. 2019;69(8):1074–8. 31431755

[77] Patel V, Patel D, Browning T, Patel S, McGurk M, Sassoon I, Guerrero Urbano T, Fenlon M. Presenting pre-radiotherapy dental status of head and neck cancer patients in the novel radiation era. Br Dent J. 2020;228(6):435–40. 10.1038/s41415-020-1327-y. 32221447 10.1038/s41415-020-1327-y

[78] Marciani RD, Ownby HE. Treating patients before and after irradiation. J Am Dent Association. 1992;123(2):108–12. 10.14219/jada.archive.1992.0052 1531839

[79] Hommez GM, De Meerleer GO, De Neve WJ, De Moor RJ. Effect of radiation dose on the prevalence of apical periodontitis–a dosimetric analysis. clin oral investig. 2012;16(6):1543–7. 10.1007/s00784-011-0665-1. 22219024 10.1007/s00784-011-0665-1

[80] Farkkila E, Rautemaa-Richardson R, Farkkila A, Gronholm L, Lauhio A. Evaluation of risk factors for oral infection with potential for spread in a 1-year cohort study. clin oral investig. 2019;23(2):905–11. 10.1007/s00784-018-2518-7. 29948280 10.1007/s00784-018-2518-7

[81] Hong CH, Napeñas JJ, Hodgson BD, Stokman MA, Mathers-Stauffer V, Elting LS, Spijkervet FK, Brennan MT. A systematic review of dental disease in patients undergoing cancer therapy. support care cancer. 2010;18(8):1007–21. 10.1007/s00520-010-0873-2. 20449756 10.1007/s00520-010-0873-2 PMC2914291

[82] Llory H, Dammron A, Gioanni M, Frank R. Some population changes in oral anaerobic microorganisms, Streptococcus mutans and yeasts following irradiation of the salivary glands. caries res. 1972;6(4):298–311. 4505878 10.1159/000259809

[83] Schwarz E, Chiu GK, Leung WK. Oral health status of Southern Chinese following head and neck irradiation therapy for nasopharyngeal carcinoma. J Dent. 1999;27(1):21–8. 9922608 10.1016/s0300-5712(98)00024-4

[84] Epstein JB, Stevenson-Moore P. Periodontal disease and periodontal management in patients with cancer. oral oncol. 2001;37(8):613–9. 10.1016/s1368-8375(01)00025-2. 11590070 10.1016/s1368-8375(01)00025-2

[85] Chronopoulos A, Zarra T, Ehrenfeld M, Otto S. Osteoradionecrosis of the jaws: definition, epidemiology, staging and clinical and radiological findings. A concise review. Int Dent J. 2018;68(1):22–30. 10.1111/idj.12318. 28649774 10.1111/idj.12318 PMC9378891

[86] Marques MA, Dib LL. Periodontal changes in patients undergoing radiotherapy. J Periodontol. 2004;75(9):1178–87. 10.1902/jop.2004.75.9.1178. 15515331 10.1902/jop.2004.75.9.1178

[87] Leung W, Jin L, Samaranayake L, Chlu G. Subgingival microbiota of shallow periodontal pockets in individuals after head and neck irradiation. oral microbiol immunol. 1998;13(1):1–10. 9573815 10.1111/j.1399-302x.1998.tb00743.x

[88] Bhandari S, Soni BW, Bahl A, Ghoshal S. Radiotherapy-induced oral morbidities in head and neck cancer patients. spec care dentist. 2020;40(3):238–50. 10.1111/scd.12469. 32378765 10.1111/scd.12469

[89] Raber-Durlacher JE, Epstein JB, Raber J, van Dissel JT, van Winkelhoff AJ, Guiot HF, van der Velden U. Periodontal infection in cancer patients treated with high-dose chemotherapy. support care cancer. 2002;10(6):466–73. 10.1007/s00520-002-0346-3. 12353125 10.1007/s00520-002-0346-3

[90] Tranfić Duplančić M, Pecotić R, Lušić Kalcina L, Pavlinac Dodig I, Valić M, Roguljić M, et al. Salivary parameters and periodontal inflammation in obstructive sleep Apnoea patients. Sci Rep. 2022;12(1):19387. 10.1038/s41598-022-23957-5. 36371504 10.1038/s41598-022-23957-5 PMC9653442

[91] Balcarcel NB, Ossola CA, Troncoso GR, Rodas JA, Astrauskas JI, Bozzini C, Elverdin JC, Fernández Solari J. Periodontal status and mandibular biomechanics in rats subjected to hyposalivation and periodontitis. Acta Odontol Latinoam. 2024;37(1):45–58. 10.54589/aol.37/1/45. 38920126 10.54589/aol.37/1/45 PMC11212216

[92] Ammajan RR, Joseph R, Rajeev R, Choudhary K, Vidhyadharan K. Assessment of periodontal changes in patients undergoing radiotherapy for head and neck malignancy: a hospital-based study. J cancer res ther. 2013;9(4):630–7. 10.4103/0973-1482.126461. 24518708 10.4103/0973-1482.126461

[93] Pischon N, Heng N, Bernimoulin JP, Kleber BM, Willich SN, Pischon T. Obesity, inflammation, and periodontal disease. J Dent Res. 2007;86(5):400–9. 10.1177/154405910708600503. 17452558 10.1177/154405910708600503

[94] Kumar S, Dagli RJ, Dhanni C, Duraiswamy P. Relationship of body mass index with periodontal health status of green marble mine laborers in kesariyaji, India. Braz Oral Res. 2009;23(4):365–9. 10.1590/s1806-83242009000400003. 20027441 10.1590/s1806-83242009000400003

[95] Bhardwaj VK, Sharma D, Jhingta P, Fotedar S, Sahore M, Manchanda K. Assessment of relationship between body mass index and periodontal status among state government employees in shimla, himachal pradesh. J Int Soc Prev Community Dent. 2013;3(2):77–80. 10.4103/2231-0762.122439. 24778984 10.4103/2231-0762.122439 PMC4000916

[96] Venkat M, Janakiram C. Association between body mass index and severity of periodontal disease among adult South Indian population: A Cross-sectional study. Indian J Community Med. 2023;48(6):902–8. 10.4103/ijcm.ijcm_148_22. 38249689 10.4103/ijcm.ijcm_148_22 PMC10795866

[97] Harris J, Rajasekar A. Correlation between body mass index and periodontitis: A clinical and biochemical analysis. cureus. 2024;16(6):e62279. 10.7759/cureus.62279. 39006613 10.7759/cureus.62279 PMC11246176

[98] Radoi L, Paget-Bailly S, Cyr D, Papadopoulos A, Guida F, Tarnaud C, et al. Body mass index, body mass change, and risk of oral cavity cancer: results of a large population-based case-control study, the icare study. cancer causes control. 2013;24(7):1437–48. 10.1007/s10552-013-0223-z. 23677332 10.1007/s10552-013-0223-z

[99] Gama RR, Song Y, Zhang Q, Brown MC, Wang J, Habbous S, et al. Body mass index and prognosis in patients with head and neck cancer. head neck. 2017;39(6):1226–33. 10.1002/hed.24760. 28323362 10.1002/hed.24760

[100] Kim CS, Park JO, Nam IC, Park SJ, Lee DH, Kim HB, Han KD, Joo YH. Associations of body mass index and waist circumference with the risk of head and neck cancer: a national population-based study. cancers (Basel). 2022;14(16). 10.3390/cancers14163880. 10.3390/cancers14163880 PMC9405652 36010881

[101] Gaudet MM, Olshan AF, Chuang SC, Berthiller J, Zhang ZF, Lissowska J, et al. Body mass index and risk of head and neck cancer in a pooled analysis of case-control studies in the international head and neck cancer epidemiology (inhance) consortium. int J Epidemiol. 2010;39(4):1091–102. 10.1093/ije/dyp380. 20123951 10.1093/ije/dyp380 PMC2929351

